# Association between Insertion-Deletion Polymorphism of the Angiotensin-Converting Enzyme Gene and Treatment Response to Antipsychotic Medications: A Study of Antipsychotic-Naïve First-Episode Psychosis Patients and Nonadherent Chronic Psychosis Patients

**DOI:** 10.3390/ijms232012180

**Published:** 2022-10-12

**Authors:** Sergej Nadalin, Sanja Dević Pavlić, Vjekoslav Peitl, Dalibor Karlović, Lena Zatković, Smiljana Ristić, Alena Buretić-Tomljanović, Hrvoje Jakovac

**Affiliations:** 1 Department of Psychiatry, General Hospital “Dr. Josip Benčević”, 35000 Slavonski Brod, Croatia; 2 School of Medicine, Catholic University of Croatia, 10000 Zagreb, Croatia; 3 Department of Medical Biology and Genetics, Faculty of Medicine, University of Rijeka, 51000 Rijeka, Croatia; 4 Department of Psychiatry, Sestre Milosrdnice University Hospital Center, 10000 Zagreb, Croatia; 5 Hospital Pharmacy, Clinical Hospital Center Rijeka, 51000 Rijeka, Croatia; 6 Department of Physiology, Immunology and Pathophysiology, Faculty of Medicine, University of Rijeka, 51000 Rijeka, Croatia

**Keywords:** angiotensin-converting enzyme (ACE), antipsychotic medication, polymorphism, insertion/deletion, treatment response

## Abstract

We investigated whether a functional insertion/deletion (I/D) polymorphism of angiotensin-converting enzyme (ACE) influenced antipsychotic treatment. At baseline, and after 8 weeks of treatment with various antipsychotic medications, we assessed patients’ Positive and Negative Syndrome Scale (PANSS) scores, PANSS factors, and metabolic-syndrome-related parameters (fasting plasma lipid and glucose levels, and body mass index). A total of 186 antipsychotic-naïve first-episode psychosis patients or nonadherent chronic psychosis individuals (99 males and 87 females) were genotyped by polymerase chain reaction analysis. The ACE-I/D polymorphism was significantly associated with changes in PANSS psychopathology only (*p* < 0.05). Compared to ACE-II homozygous males, ACE-DD homozygous and ACE-ID heterozygous males manifested significantly greater decreases in PANSS positive score, PANSS excitement factor, and PANSS cognitive factor. ACE-DD homozygous females manifested higher decreases in PANSS depression factor compared to ACE-II homozygous and ACE-ID heterozygous females. The polymorphism’s effect size was estimated as moderate to strong, while its contribution to the PANSS psychopathology ranged from ~5.4 to 8.7%, with the lowest contribution observed for PANSS positive score changes and the highest for PANSS depressive factor changes. Our results indicate that ACE-I/D polymorphism had a statistically significant but weak gender-specific impact on psychopathology data, and showed no association between ACE-I/D polymorphism and metabolic-syndrome-related parameters.

## 1. Introduction

Angiotensin-converting enzyme (ACE) catalyzes the conversion of angiotensin I to angiotensin II in the renin–angiotensin system, and inactivates bradykinin via the kinin–kallikrein system [1,2]. A functional 287-base-pair ACE-insertion/deletion (I/D) polymorphism is the most well-studied genetic alteration that influences ACE expression in humans. ACE-DD homozygous individuals exhibit the highest ACE plasma activity, ACE-II homozygous individuals show the lowest activity, and those who are ACE-ID heterozygous exhibit intermediate activity [3,4]. ACE and angiotensin II have been suggested to play important roles in brain dopaminergic neurotransmission [5,6,7]; therefore, the ACE-I/D polymorphism has been investigated in schizophrenia etiology and clinical expression, as assessed using the Positive and Negative Syndrome Scale (PANSS) [4,8,9,10,11,12,13].

We previously investigated whether the ACE-I/D polymorphism might influence schizophrenia risk and severity of PANSS psychopathology among chronic patients taking antipsychotic medication, from the Croatian population [8]. While we did not find evidence that the ACE-I/D polymorphism was associated with elevated schizophrenia risk, we did observe several significant associations between the ACE-I/D polymorphism and PANSS scores. Specifically, we found that patients carrying the D allele (ACE-DD homozygous and ACE-ID heterozygous) manifested higher negative and general psychopathology PANSS scores, compared to patients who were ACE-II homozygous. Additionally, after adjustment for sex, we found that the presence of the D allele in the ACE genotype contributed to increased general PANSS scores among males with schizophrenia.

We also investigated whether ACE-I/D polymorphism might influence plasma lipid and glucose levels among schizophrenia patients receiving antipsychotic treatment [14]. Schizophrenia patients (specifically those taking antipsychotic medication) are more likely to exhibit weight gain, lipid disturbance, and glucose dysregulation, which makes them an interesting group in which to study the genetic basis of dyslipidemia and diabetes [15,16]. Furthermore, among individuals from the general population, ACE-I/D polymorphism has been extensively linked to dyslipidemia, diabetes, and metabolic syndrome [17,18,19,20]. We found that the ACE-I/D polymorphism had an intriguing gender-specific effect on the investigated metabolic parameters. Specifically, we found that ACE-ID heterozygous females exhibited significantly higher glucose levels compared to ACE-DD homozygous and ACE-II homozygous females. On the other hand, males carrying the D allele (ACE-DD homozygous and ACE-ID heterozygous) tended to show elevated triglyceride levels compared to ACE-II homozygous males. To our knowledge, no other published study has addressed the potential association between ACE-I/D polymorphism and metabolic-syndrome-related parameters among schizophrenia patients.

Only sparse data are available regarding the potential association between ACE-I/D polymorphism and antipsychotic treatment. In one study among antipsychotic-naïve first-episode psychosis patients from the Brazilian population, researchers investigated whether ACE-I/D polymorphism-associated changes in plasma ACE activity upon treatment with the antipsychotic risperidone might influence symptom improvement, as measured by total PANSS scores [4]. Indeed, that group had previously reported increased ACE activity in chronic schizophrenia patients compared to healthy controls, and suggested the potential of using combined ACE genotype and activity for predicting schizophrenia [10]. Their current findings indicate that risperidone treatment significantly increased the ACE activity among ACE-DD homozygous patients, while only a trend of increased ACE activity was observed among ACE-ID heterozygous and ACE-II homozygous individuals. However, their study demonstrated no significant correlation between increases of ACE activity and symptom improvement [4].

Available data indicate that antipsychotic treatment increases ACE activity [4], and our previous work suggests the potential relevance of ACE-I/D polymorphism in the severity of PANSS psychopathology and metabolic-syndrome-related parameters, among chronic schizophrenia patients taking antipsychotic medication [8,14]. Therefore, in the present study we hypothesized that ACE-I/D polymorphism might influence antipsychotic treatment-related changes in PANSS psychopathology, plasma lipid and glucose levels, and BMI values. Many findings indicate that determining PANSS factors provides a more robust understanding of the structure of schizophrenia symptom change upon antipsychotic treatment [21,22]. Accordingly, here we assessed PANSS psychopathology by determining PANSS scores plus PANSS factors. Based on our previous observation of gender-specific differences in the effects of ACE-I/D polymorphism in schizophrenia [8,14,23]—and similar findings in other diseases/conditions, such as multiple sclerosis [24] and lung cancer [25]—we performed separate analyses for male and female patients. This is the first study to investigate the association of ACE-I/D polymorphism with metabolic-syndrome-related parameters upon antipsychotic treatment. Our sample comprised antipsychotic-naïve first-episode psychosis patients and nonadherent chronic psychosis patients who were treated for 8 weeks with various, mainly atypical, antipsychotic medications.

## 2. Results

Males and females significantly differed in age, age of onset, plasma total cholesterol, HDL cholesterol, triglyceride levels, and BMI values (Table 1). Specifically, compared to females, males exhibited lower age, earlier onset, lower HDL cholesterol levels, higher total cholesterol, higher triglycerides, and higher BMI values (*p* < 0.01 and *p* < 0.05). For both genders, total cholesterol, LDL cholesterol, HDL cholesterol, triglyceride, and glucose levels were within the reference range [26]. Females had BMI values within the normal range, while males were slightly in the overweight range [27,28]. 

The allele and genotype frequencies of the ACE-I/D polymorphism were similar to those reported previously [8,14,23], and did not significantly deviate from that reported in the overall European population [29]. The ACE genotype distributions among males and females were consistent with Hardy–Weinberg equilibrium, and the genotype and allele distributions did not significantly differ between genders (Table 2).

The ACE-I/D polymorphism was significantly associated with changes in PANSS psychopathology only (Table 3). Specifically, compared to ACE-II homozygous males, males positive for the ACE-D allele (ACE-DD homozygous and ACE-ID heterozygous) exhibited significantly greater decreases in PANSS positive score (−8.7 ± 9.5 vs. −1.0 ± 12.4, z = −1.96, *p* = 0.049, Cohen’s *d* = 0.78), PANSS excitement factor (−4.3 ± 2.7 vs. −2.0 ± 1.8, z = −2.62, *p* = 0.007, Cohen’s *d* = 0.89), and PANSS cognitive factor (−2.0 ± 1.6 vs. −0.9 ± 1.4, z = −2.21, *p* = 0.030, Cohen’s *d* = 0.70). On the other hand, ACE-DD homozygous females exhibited greater decreases in PANSS depression factor compared to ACE-II homozygous and ACE-ID heterozygous females: −8.4 ± 2.8 vs. −6.6 ± 2.8, z = −2.53, *p* = 0.010, Cohen’s *d* = 0.64. 

Among males, the ACE-D allele significantly predicted changes in PANSS positive symptom score (β = −0.23, *p* = 0.035), and PANSS excitement factor (β = −0.26, *p* = 0.017). Among females, the ACE-I allele appeared as a significant predictor of changes in PANSS depression factor (β = 0.30, *p* = 0.008) (Table 4). In males, changes in PANSS cognitive factor were significantly predicted by the number of psychotic episodes only (β = 0.25, *p* = 0.023). Compared to ACE-II homozygous males, among ACE-DD homozygous and ACE-ID heterozygous males, negative β values for PANSS positive symptom score and PANSS excitement factor indicated higher decreases of these variables. Compared to ACE-II homozygous and ACE-ID heterozygous females, among ACE-DD homozygous females, positive β values for PANSS depression factor indicated greater decreases of this variable. The contribution of ACE-I/D polymorphism to PANSS psychopathology ranged from ~5.4% to 8.7%, with the lowest contribution observed for PANSS positive score changes (R^2^ change = 0.054) and the highest for PANSS depressive factor changes (R^2^ change = 0.087) (Table 4).

## 3. Discussion

The primary finding of the current study is that ACE-I/D polymorphism influence on changes in PANSS psychopathology upon antipsychotic treatment. We found no association between ACE-I/D polymorphism and metabolic-syndrome-related parameters in male or female patients. Specifically, our results suggest that compared to ACE-II homozygous males, males positive for the ACE-D allele (ACE-DD homozygous and ACE-ID heterozygous) exhibit greater improvement in PANSS positive symptom score and PANSS excitement factor. On the other hand, ACE-DD homozygous females exhibited greater improvement in PANSS depression factor, compared to females positive for the ACE-I allele (ACE-II homozygous and ACE-ID heterozygous) (Table 3 and Table 4). Although the contribution of ACE-I/D polymorphism to PANSS psychopathology was weak (~5.4% to 8.7%), the Cohen’s *d* values indicated that the polymorphism had moderate-to-strong effect sizes (0.64 to 0.89). 

Our current findings indicate that the ACE-D allele had a protective effect towards the improvement of clinical psychopathology upon antipsychotic treatment. In contrast, our previous findings among chronic schizophrenia patients under antipsychotic treatment suggested that patients positive for the ACE-D allele (ACE-DD homozygous and ACE-ID heterozygous) exhibited greater severity of PANSS psychopathology compared to ACE-II homozygous patients [8]. Moreover, our current findings indicated no association between ACE-I/D polymorphism and changes in metabolic-syndrome-related parameters upon antipsychotic treatment (Table 3). In contrast, our previous data suggest that ACE-I/D polymorphism might influence plasma glucose and lipid levels among chronic patients under antipsychotic treatment [14]. Importantly, in our previous work, PANSS psychopathology and metabolic-syndrome-related parameters were assessed at a single point, during hospital admission due to clinical deterioration, and we did not consider whether patients were adherent to antipsychotic medications. Additionally, in our previous work, when assessing PANSS psychopathology, we only determined PANSS scores, while PANSS scores and PANSS factors were also considered in the current study [8,14]. The favorable effect of the ACE-DD homozygous genotype on PANSS depression factor upon antipsychotic treatment, which was observed among female patients in the current study, might be explained by data previously reported by Nani et al. [4], indicating that risperidone treatment increases ACE activity, mainly among ACE-DD homozygous individuals (Table 3 and Table 4). In this context, the presence of ACE-DD homozygous genotypes may have contributed to the improvements of PANSS positive symptom score and PANSS factor upon antipsychotic treatment among males positive for the ACE-D allele. Intriguingly, Nani et al. [4] reported that ACE-I/D polymorphism-associated increases in plasma ACE activity were not correlated with symptom improvement in their study. Therefore, other yet-undefined factors may affect the relationship between ACE-I/D polymorphism and response to antipsychotic treatment. For instance, noncanonical ACE substrates, such as substance P and neurotensin, have been shown to play important roles in dopaminergic neurotransmission and response to antipsychotic treatment [30,31,32,33]. 

Several findings may explain the gender-specific differences in ACE-I/D polymorphism observed in the current study [34,35,36,37,38]. Estrogen replacement therapy has been associated with genotype-associated changes in ACE activity among postmenopausal women [34,35]. One study showed significantly reduced plasma ACE activity in women with the ACE-ID and ACE-II genotypes [34], and another study showed ACE reductions in women with the ACE-ID and ACE-DD genotypes [35]. Furthermore, estrogen reportedly exerts favorable effects on brain dopaminergic neurotransmission: estrogen increases dopamine synthesis; decreases dopamine degradation, reuptake, and recapture; and upregulates dopaminergic receptors [36,37,38].

Our results suggest that compared to PANSS scores, PANSS factors indicate more subtle changes in PANSS psychopathology upon antipsychotic treatment (Table 3 and Table 4). This finding is consistent with previous reports that the determination of PANSS factors may enable more sensitive assessment of how gene polymorphisms affect therapeutic outcomes among psychotic patients [39,40]. Indeed, it has been established that a change in PANSS factor structure does not necessarily indicate a flaw in the PANSS scores [21,22]. Moreover, the PANSS factor structure might also reflect latent dimensions of psychosis and/or other mental illnesses [22,41].

We speculate that ACE-I/D polymorphism, by affecting ACE activity, might directly influence dopamine homeostasis and contribute to changes in PANSS psychopathology without peripheral metabolic changes. Interestingly, many studies indicate that polymorphisms of the genes encoding dopamine and other neurotransmitter receptors are frequently associated with metabolic changes, regardless of antipsychotic treatment [42,43]. Accordingly, we further speculate that altered ACE activity might affect neuronal signaling, even without changes in the neurotransmitter–receptor axis [44,45].

The limitations of our study include the relatively small sample, which leaves open the possibility that some effects were not detected. Additionally, nonadherence to antipsychotic medications was assessed via anamnestic information, and we only assessed a single polymorphism of the ACE gene. Furthermore, in contrast to the previous study assessing the role of ACE-I/D polymorphism in response to risperidone [4], our study comprised patients who were rather heterogeneous regarding antipsychotic medications. Nevertheless, the use of various antipsychotic medications has been shown to affect ACE activity, and there is evidence of both increased ACE activity [46,47] and decreased ACE activity [48] among patients treated with these medications.

In conclusion, the current data indicate that ACE-I/D polymorphism had a statistically significant but weak gender-specific impact on changes of PANSS psychopathology data upon antipsychotic treatment. We also found no association between ACE-I/D polymorphism and metabolic-syndrome-related parameters in male or female patients.

## 4. Materials and Methods

We recruited a total of 186 antipsychotic-naïve first-episode psychosis patients or nonadherent chronic psychosis individuals. All participants were Croatian citizens, who were treated at the Department of Psychiatry in the University Hospital Center Sestre Milosrdnice, Zagreb between 2016 and 2021. Each patient gave written informed consent. This study was approved by the appropriate Ethics Committee, and was conducted in accordance with the ethical standards expressed in the latest version of the Declaration of Helsinki. Diagnoses were assessed by at least two psychiatrists, according to the Diagnostic and Statistical Manual of Mental Disorders (DSM-V) criteria, using a structured clinical interview. Among the participants, 163 (87.7%) were diagnosed with schizophrenia, 9 (4.8%) with schizoaffective disorder, and 14 (7.5%) with psychotic disorder not otherwise specified.

Antipsychotic-naïve patients had never previously been treated with antipsychotic medications. Nonadherent chronic patients were—according to auto-anamnestic and hetero-anamnestic information—non-compliant with their antipsychotic medication usage, or had been off antipsychotic depot injections for at least 1 month. Both patient groups received one or more of the following medications for 8 weeks: clozapine (*n* = 84), risperidone (*n* = 45), aripiprazole (*n* = 45), paliperidone (*n* = 40), aripiprazole depo (*n* = 28), haloperidol (*n* = 28), fluphenazine (*n* = 14), and olanzapine (*n* = 13). When anxiolytic or hypnotic medications were not sufficiently effective, the antipsychotic promazine was also prescribed to many patients (*n* = 64), usually at smaller doses (75 to 150 mg per day). Antipsychotic-naïve first-episode patients and nonadherent chronic patients presented with similar PANSS psychopathology data, and did not significantly differ in most metabolic-syndrome-related parameters (*p* > 0.05). Therefore, we combined these two groups of participants for statistical analyses. Table 1 presents the patients’ characteristics at baseline according to gender.

Age at onset was defined as the patient’s age at their first hospital admission due to a psychotic episode, at which the diagnosis of schizophrenia was used. Evaluation of PANSS psychopathology at baseline was performed during a psychotic state of the illness requiring hospitalization. PANSS scales were divided into five symptom factors: positive (P1, P3, P6, and G9), negative (N2, N3, N4, N6, and G7), excitement (P4, P7, and G1), depression (G2, G3, and G6), and cognitive (G10 and G12) [49,50,51]. Fasting plasma total cholesterol, HDL cholesterol, LDL cholesterol, triglycerides, and glucose levels were measured using an Abbott Architect c8000 analyzer, at the Department of Clinical Chemistry in the University Hospital Center Sestre Milosrdnice, Zagreb. The following values were considered elevated: total cholesterol > 5.0 mmol/L, LDL > 3.0 mmol/L, triglycerides > 2.0 mmol/L, and glucose > 6.1 mmol/L, while HDL < 1.0 mmol/L was considered decreased [26]. BMI was calculated as weight (kg) divided by height squared (m^2^). Patients were classified as obese with BMI values > 30, or as non-obese if overweight (BMI: 25–30) or of normal body weight (BMI: 18.5–25) [27,28]. From whole blood samples, genomic DNA was extracted using the FlexiGene DNA kit 250 (QIAGEN GmbH, Hilden, Germany) according to the manufacturer’s instructions. Polymerase chain reaction-based genotyping was performed in the Department of Medical Biology and Genetics, in the Faculty of Medicine, University of Rijeka, using previously described protocols [3]. To exclude mistyping of ACE-ID heterozygotes as ACE-DD homozygotes, all ACE-DD genotypes were confirmed using insertion-specific PCR [52].

### Statistical Analyses

Statistical analyses were conducted using Statistica for Windows, version 12 (StatSoft, Inc., Tulsa, OK, USA). To compare the characteristics of male and female patients, we used nonparametric Mann–Whitney *U* tests or chi-square (χ^2^) tests. We used Mann–Whitney *U* tests to examine associations between ACE-I/D polymorphism and changes in mean PANSS psychopathology data, plasma lipid and glucose levels, and BMI values after antipsychotic treatment. The Cohen’s *d* method was used to calculate the standardized effect size [53]. Associations that appeared significant (*p* < 0.05) were further examined by multiple regression analyses, with adjustment for age and number of psychotic episodes. 

## Figures and Tables

**Table 1:** Patients’ characteristics at baseline.

	Males (*N* = 99)	Females (*N* = 87)	*p*
Antipsychotic-naïve first-episode patients/nonadherent chronic patients	41/58	26/61	0.102
Age, years	31.9 ± 11.7	41.6 ± 14.9	**<0.001**
Age of onset, years	25.6 ± 8.3	32.1 ± 12.3	**<0.001**
Number of psychotic episodes	2.7 ± 2.0	3.1 ± 2.0	0.131
PANSS positive symptom score	22.4 ± 6.4	22.0 ± 5.8	0.884
PANSS negative symptom score	26.6 ± 7.0	26.2 ± 8.0	0.674
PANSS general psychopathology score	50.8 ± 9.0	49.2 ± 8.7	0.238
PANSS total symptom score	99.8 ± 17.7	97.4 ± 18.4	0.434
PANSS positive factor	13.3 ± 4.1	12.9 ± 3.8	0.445
PANSS negative factor	19.0 ± 5.2	18.9 ± 6.3	0.833
PANSS excitement factor	8.4 ± 2.7	8.2 ± 2.7	0.645
PANSS depression factor	9.6 ± 2.7	9.1 ± 3.0	0.260
PANSS cognitive factor	5.9 ± 1.8	5.8 ± 2.1	0.731
Total cholesterol, mmol/L	4.6 ± 1.0	4.9 ± 1.1	**0.032**
LDL cholesterol, mmol/L	2.7 ± 0.8	3.0 ± 1.0	0.091
HDL cholesterol, mmol/L	1.1 ± 0.3	1.3 ± 0.4	**<0.001**
Triglycerides, mmoL/L	1.5 ± 1.1	1.2 ± 0.5	**0.024**
Glucose, mmol/L	5.6 ± 1.6	5.5 ± 1.0	0.878
Body mass index, kg/m^2^	25.6 ± 4.7	23.8 ± 4.2	**0.011**

Differences were compared using the Mann–Whitney *U* test, with the exception of antipsychotic-naïve first-episode patients/nonadherent chronic patients ratio (χ^2^ test). PANSS indicates Positive and Negative Syndrome Scale.

**Table 2:** The frequency of ACE genotypes and alleles among male and female patients.

	Genotypes (%)			Alleles (%)	
	DD	ID	II	D	I
Males (*N* = 99) ^a^	28 (28.3)	57 (57.6)	14 (14.1)	113 (57.1)	83 (42.9)
Females (*N* = 87) ^b^	26 (30.2)	50 (57.0)	11 (12.8)	102 (58.6)	72 (41.4)
	χ^2^ = 0.12, df = 2, *p* = 0.942			χ^2^ = 0.04, df = 1, *p* = 0.851	

^a^ Hardy–Weinberg: χ^2^ = 3.03, *p* = 0.082; ^b^ Hardy–Weinberg: χ^2^ = 2.97, *p* = 0.085.

**Table 3:** PANSS psychopathology data and metabolic-syndrome-related parameters upon antipsychotic treatment according to ACE-I/D polymorphism.

	Males (*N* = 99)	Mean ± SD	z	*p*	Females (*N* = 87)	Mean ± SD	z	*p*
PANSS positive symptom score	DD, ID II	−8.6 ± 9.5 −1.0 ± 12.4	−1.96	**0.049**	DD, ID II	−9.0 ± 8.9 −7.0 ± 7.5	−1.11	0.270
	II, ID DD	−8.2 ± 9.4 −7.1 ± 11.5	−0.18	0.858	II, ID DD	−8.1 ± 9.8 −10.1 ± 5.5	−0.68	0.502
PANSS negative symptom score	DD, ID II	−6.4 ± 9.5 −6.8 ± 11.0	−0.17	0.864	DD, ID II	−7.8 ± 8.3 −6.2 ± 11.7	−0.09	0.926
	II, ID DD	−6.6 ± 9.1 −6.0 ± 10.8	−0.11	0.909	II, ID DD	−7.5 ± 9.8 −7.9 ± 5.6	−0.21	0.837
PANSS general psychopathology score	DD, ID II	−13.5 ± 18.2 −9.4 ± 18.5	−0.71	0.479	DD, ID II	−14.9 ± 13.7 −8.3 ± 19.2	−1.10	0.296
	II, ID DD	−12.5 ± 16.7 −14.5 ± 21.4	1.34	0.180	II, ID DD	−12.9 ± 16.0 −16.6 ± 10.1	−1.33	0.185
PANSS total symptom score	DD, ID II	−28.2 ± 4.7 −17.2 ± 34.1	−1.19	0.235	DD, ID II	−31.7 ± 28.3 −21.5 ± 37.4	−0.89	0.376
	II, ID DD	−27.2 ± 31.9 −26.6 ± 40.5	0.30	0.765	II, ID DD	−28.5 ± 33.2 −34.6 ± 18.8	−1.14	0.255
PANSS positive factor	DD, ID II	−6.4 ± 3.3 −5.6 ± 6.6	−0.24	0.810	DD, ID II	−5.9 ± 3.9 −6.1 ± 3.5	−0.24	0.813
	II, ID DD	−6.2 ± 4.0 −6.6 ± 3.3	0.30	0.767	II, ID DD	−5.7 ± 3.9 −6.2 ± 3.8	−0.86	0.391
PANSS negative factor	DD, ID II	−6.7 ± 4.2 −7.2 ± 5.9	0.24	0.810	DD, ID II	−6.8 ± 5.7 −7.0 ± 4.7	−0.03	0.971
	II, ID DD	−6.8 ± 4.4 −6.7 ± 4.5	−0.20	0.837	II, ID DD	−7.2 ± 5.8 −6.2 ± 5.0	0.25	0.797
PANSS excitement factor	DD, ID II	−4.3 ± 2.7 −2.0 ± 1.8	−2.62	**0.007**	DD, ID II	−3.7 ± 2.9 −2.5 ± 2.7	−1.90	0.059
	II, ID DD	−4.1 ± 2.8 −3.9 ± 2.4	0.09	0.928	II, ID DD	−3.5 ± 3.1 −3.7 ± 2.2	−0.54	0.591
PANSS depression factor	DD, ID II	−8.0 ± 2.6 −7.6 ± 2.0	−0.41	0.686	DD, ID II	−7.3 ± 2.9 −6.3 ± 2.7	−0.88	0.384
	II, ID DD	−7.6 ± 2.3 −8.6 ± 2.8	1.71	0.092	II, ID DD	−6.6 ± 2.8 −8.4 ± 2.8	−2.53	**0.010**
PANSS cognitive factor	DD, ID II	−2.0 ± 1.6 −0.9 ± 1.4	−2.21	**0.030**	DD, ID II	−1.7 ± 1.9 −1.0 ± 1.4	−0.96	0.353
	II, ID DD	−1.7 ± 1.6 −2.2 ± 1.7	1.26	0.222	II, ID DD	−1.7 ± 2.1 −1.5 ± 1.2	0.04	0.967
Total cholesterol, mmol/L	DD, ID II	0.5 ± 0.8 0.0 ± 0.4	1.60	0.112	DD, ID II	0.4 ± 0.9 −0.0 ± 0.6	1.34	0.182
	II, ID DD	0.4 ± 0.8 0.5 ± 0.7	−0.91	0.367	II, ID DD	0.3 ± 0.9 0.5 ± 0.9	0.93	0.350
LDL cholesterol, mmol/L	DD, ID II	0.3 ± 0.6 0.1 ± 0.2	0.93	0.354	DD, ID II	0.2 ± 0.7 0.1 ± 0.6	0.36	0.718
	II, ID DD	0.3 ± 0.6 0.2 ± 0.6	0.59	0.558	II, ID DD	0.1 ± 0.6 0.3 ± 0.8	1.60	0.109
HDL cholesterol, mmol/L	DD, ID II	0.1 ± 0.3 0.0 ± 0.3	0.40	0.687	DD, ID II	0.1 ± 0.3 0.0 ± 0.3	0.39	0.706
	II, ID DD	−0.0 ± 0.3 0.1 ± 0.3	−1.23	0.219	II, ID DD	0.1 ± 0.3 0.1 ± 0.3	0.94	0.356
Triglycerides, mmoL/L	DD, ID II	0.3 ± 1.0 0.1 ± 0.8	0.29	0.774	DD, ID II	0.4 ± 1.0 −0.1 ± 0.6	1.10	0.276
	II, ID DD	0.2 ± 1.0 0.4 ± 0.8	−1.40	0.161	II, ID DD	0.4 ± 1.2 0.2 ± 0.6	−0.49	0.619
Glucose, mmol/L	DD, ID II	0.2 ± 1.7 0.1 ± 1.0	−0.35	0.729	DD, ID II	0.2 ± 1.6 −0.5 ± 1.1	1.37	0.169
	II, ID DD	0.3 ± 1.8 −0.0 ± 1.0	0.49	0.626	II, ID DD	0.2 ± 1.8 −0.1 ± 1.0	0.10	0.921
Body mass index, kg/m^2^	DD, ID II	0.6 ± 2.0 −0.8 ± 2.2	1.86	0.062	DD, ID II	0.7 ± 1.9 0.8 ± 1.5	−0.16	0.872
	II, ID DD	0.6 ± 1.6 0.1 ± 2.8	0.19	0.846	II, ID DD	0.7 ± 2.0 0.7 ± 1.6	0.10	0.912

Differences were compared using Mann–Whitney *U* test. PANSS indicates Positive and Negative Syndrome Scale. Changes were compared using Mann–Whitney *U* test. PANSS indicates Positive and Negative Syndrome Scale.

**Table 4:** PANSS psychopathology data after antipsychotic treatment, predicted by ACE-I/D polymorphism.

Males (*N* = 99)					
Dependent Variable	Predictor	β	R^2^ Change	F	*p*
PANSS positive symptom score	ACE-D allele	−0.23	0.054	4.59	**0.035**
PANSS excitement factor	ACE-D allele	−0.26	0.067	5.93	**0.017**
PANSS cognitive factor	Number of psychotic episodes	0.25	0.062	5.39	**0.023**
**Females (*N* = 87)**					
**Dependent Variable**	**Predictor**	**β**	**R^2^ Change**	**F**	***p***
PANSS depression factor	ACE-I allele	0.30	0.087	7.50	**0.008**

Candidate predictor variables included ACE-D allele, ACE-I allele, age, and number of psychotic episodes. Criteria used for predictor variable’s entry or removal: F = 3 to enter, F = 1 to remove.

## Data Availability

The data presented in this study are available on request from the corresponding author if data sharing is approved by ethics committee. The data are not publicly available due to data protection laws and adherence to ethical principles.
